# Comparative study between ultra-mini-percutaneous nephrolithotomy versus stented extracorporeal shock wave lithotripsy for treatment of renal stones in Egypt

**DOI:** 10.1080/2090598X.2023.2211897

**Published:** 2023-05-16

**Authors:** Ahmed Ibrahim Radwan, Ahmed Mohsen Ibrahim Saif, Younan Ramsis Samir, Wael Ali Maged, Mohamed A. Gamal

**Affiliations:** Faculty of Medicine, Ain Shams University, Cairo, Egypt

**Keywords:** Nephrolithiasis, shock wave lithotripsy (SWL), ultra-mini-PCNL

## Abstract

**Objectives:**

The purpose of this study is to compare results, safety and outcome of ultra-mini-percutaneous nephrolithotomy (PCNL) versus stented shock wave lithotripsy (SWL) for the management of renal calculi sized 10–20 mm.

**Methods:**

This study was conducted at Urology Department, Faculty of Medicine, Ain Shams University. After meeting inclusion and exclusion criteria, 90 patients were randomized to either ultra-mini-PCNL group or stented SWL group through the closed-envelope technique, with 45 patients in each group. Patient data were collected preoperatively, immediately postoperatively and 2 and 4 weeks postoperatively assessing operative time, hospital stay, complications including haematuria, fever, the need for blood transfusion, residual stones and the need for retreatment.

**Results:**

Stone-free rate (SFR) was higher in the ultra-mini-PCNL group compared to the stented SWL group, with no statistically significant difference with *P*-value = 0.316. As for the need for retreatment, it was slightly higher in the stented SWL group compared to the ultra-mini-PCNL group, yet this difference was statistically insignificant with *P*-value = 0.681.

We found no statistically significant difference between both groups regarding post-operative complications including fever, haematuria and need for blood transfusion, respectively.

Operative time and hospital stay were significantly higher in the ultra-mini-PCNL group compared to the stented SWL group with *P*-value < 0.001 for both.

**Conclusion:**

Both stented SWL and ultra-mini-PCNL are good treatment choices for renal stones sized less than 2 cm with low complication rates. Stone size indices were significant predictor for the need for retreatment. Further studies to compare SFR based on stone size in both interventions are needed.

## Introduction

Nephrolithiasis is the third most common disease of the urinary tract after urinary tract infections (UTIs) and prostatic diseases. It also has a 1-year recurrence rate of 7% and 10-year recurrence rate of 50% [1,2].

Management of renal stones has seen a great change over the past years. The choice of the treatment modality is based upon balancing morbidity vs stone clearance. Low-risk procedure with high retreatment percentage vs another relatively higher risk procedure with lower retreatment chances [3].

With the introduction of minimally invasive techniques, shock wave lithotripsy (SWL), retrograde intrarenal surgery (RIRS) and percutaneous nephrolithotomy (PCNL) are different strategies in managing renal stones. Open, laparoscopic and robotic surgeries have their place only in highly selected cases [2].

According to the European Association of Urology (EAU) Guidelines on Urolithiasis, PCNL is a gold-standard treatment for renal stones sized >20 mm and SWL or RIRS is the first-line therapy for renal stones sized <10 mm [4].

SWL is a minimally invasive intervention with good patient tolerance; it is considered the first-line treatment for nephrolithiasis <20 mm in size. However, poor clearance of lower calyceal stone fragments due to gravity or unfavourable infundibulopelvic angle limits the efficacy of SWL for treating lower calyceal stones [5].

RIRS introduced a newly developed higher technique in exploring the upper urinary tract, permitting us to deal with any calculus regardless of its site. Using RIRS has revealed higher stone-free rate (SFR) with less complications such as haemorrhage and renal damage. On the other hand, RIRS is being challenged by the stone size, forcing the procedure to be staged, ureteric injury risk and the higher cost of its usage and maintenance that might have caused to slow its rapid diffusion [6].

Conventional PCNL (24–30 F) remains the gold-standard procedure used in managing large renal stones, although it allows reaching high SFR on the expense of the possibility of causing several complications such as bleeding, pain and a long convalescence time due to its large access tract. To minimize renal parenchymal injury related to the conventional PCNL, minimally invasive PCNL with a smaller tract size has been introduced. According to the size of the access tract, minimally invasive PCNL can be divided into mini-PCNL (14–22 Fr) and ultra-mini-PCNL (11–13 Fr) [5,7,8].

PCNL carried a significantly higher SFR than RIRS, especially for lower calyceal stones; however, mini-PCNL came at the expense of a longer hospital stay and an increased haemoglobin (Hb) drop. Although PCNL could exhibit an SFR of 93.8%, its complication rate reached 14.5% in a global survey [9].

Ultra-mini-PCNL presents a unique opportunity for hard-to-access stones, which impacted lower calyceal stones with an acute infundibular angle or stones in a calyceal diverticulum with less risk of blood loss [7].

Regarding stones in between 10 and 20 mm and according to the EAU Guidelines on Urolithiasis, SWL or any endourologic treatments are the choices of treatment with no specific preference, leaving the choice for surgeon’s preference and patient counselling [4].

The purpose of this study is to compare results, safety and outcome of ultra-mini-PCNL versus stented extracorporeal SWL for the management of renal calculi sized 10–20 mm aiming to help guide the choice of the treatment modality.

## Materials and methods

### Ethical considerations

This is a randomized clinical trial, registered at NIH ClinicalTrials.gov (http://www.clinicaltrials.gov/), number NCT05697341.

This study was reviewed and approved by the Research Ethics Committee of Faculty of Medicine, Ain Shams University, with approval number MD 42/2021.

### Patients

This study was conducted at Urology Department, Faculty of Medicine, Ain Shams University. Patients were apprenticed from the urology clinic from March 2021 till March 2022. Inclusion criteria included patients between the ages 18 and 60 years and complaining of radio-opaque renal stones ranging from 10 to 20 mm with BMI not exceeding 40.

Exclusion criteria included radiolucent stones, calculi with size smaller than 1 cm or larger than 2 cm, and patients with congenital renal anomalies or spinal deformities or BMI exceeding 40. Pregnant females or patients who had uncorrected bleeding diathesis or untreated UTI were also excluded.

Using PASS 15 program for sample size calculation based on reviewing the results of a previous study (Zhang et al. 2019), we assumed a medium effect size difference between the two groups regarding the SFR (*d* = 0.6); based on these findings, sample size of 90 patients (45 per group) achieves 80% power to reject the null hypothesis of zero effect size when the significance level (alpha) is 0.050 and the population effect size is 0.60 using a two-sided *z* test.

**Figure 1.**
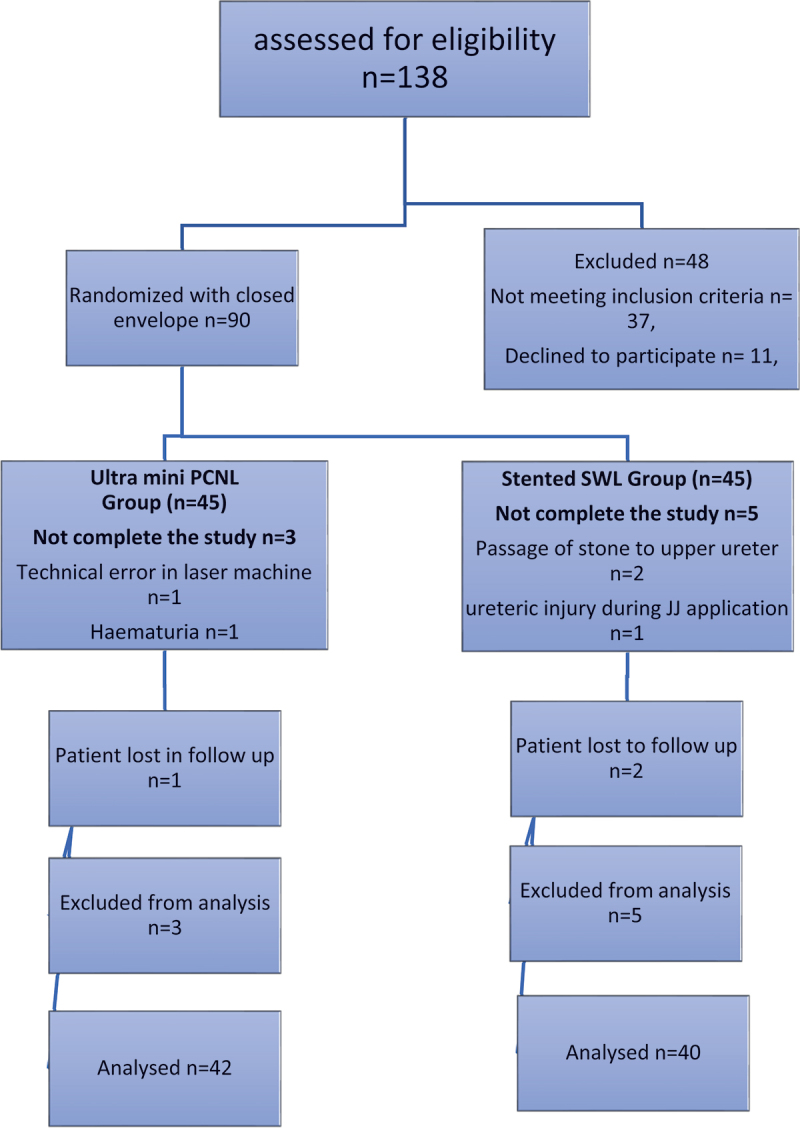
Flowchart for our study.

In this study, 138 patients were assessed for eligibility; 48 patients were excluded, 37 of which were not meeting our inclusion criteria and 11 patients declined to participate in the study as detailed in the CONSORT flowchart (Figure 1).

After meeting our inclusion and exclusion criteria, 90 patients were thoroughly informed about the study and after feeling well about participating in it a written informed consent was taken from them. Patients were randomized to either ultra-mini-PCNL group or stented SWL group through the closed-envelope technique.

### Procedure

Preoperative evaluation included careful history taking, general and local examination, urine analysis, perioperative laboratory tests, Computed tomography of the Urinary Tract (CTUT) with Hounsfield units (HU) estimation and plain X-ray of the urinary tract (KUB).

All patients were given perioperative antibiotic prophylaxis.

In the ultra-mini‑PCNL group, a 5 Fr open‑end ureteric catheter was applied in the renal pelvis and a pyelography was done in the lithotomy position after the induction of general anaesthesia. Patients were then placed in the prone position. Punctures were done in the prone position by a single consultant. The targeted calyx was punctured with a Cook diamond tip 18 G puncture needle guided by fluoroscopy using standard bull’s eye technique. Single tract dilatation method was used with One Step Dilator (11 Fr), introduced on an Alkan rod that was advanced on the guidewire, followed by its Operating Sheath (Storz Dilator and Operating Sheaths for MIP XS 7.5 Fr) under fluoroscopy guidance. Storz Nephroscope for MIP XS/S along with Swiss Lithoclast master pneumatic lithotripter was used for stone disintegration. Stone fragments were flushed out by the rapid nephroscope removal depending on the ‘vortex’ effect and by applying wash using a 6 Fr nelaton catheter through the operating sheath. Ureteric catheter was left in the patients attached to the urethral catheter as postoperative stenting and was removed on the second postoperative day.

In the stented SWL group, a 5 Fr open‑end ureteric catheter was applied in the renal pelvis and a pyelography was done in the lithotomy position after the induction of general anaesthesia. Double J stent (JJ) is applied either 5–26 or 5–28 according to the patient. Extracorporeal SWL was administered with an electromagnetic shock wave lithotripter (Siemens electromagnetic lithotripter devices). Patients were placed in the supine position with the shock head facing the patient’s back. Stone fragmentation was done and monitored using fluoroscopy guidance. Shock waves were given at a frequency of 60 shocks/min till the complete fragmentation of the stone without exceeding 3000 shocks in the session.

Follow-up of patients was done postoperatively by taking careful, detailed history and examination. Assessment of postoperative pain, fever, sepsis and haematuria was done. Follow up of postoperative serum Hb level, serum creatinine, blood urea nitrogen (BUN), sodium (Na+) and potassium (K+) was also done.

### Follow-up

Follow-up of patients was done after 2 and 4 weeks, assessing the following: careful and detailed history taking and examination, KUB, urine analysis, culture and sensitivity test, serum creatinine and BUN levels.

Stone‑free status was assessed with a KUB at 2 or 4 weeks after ultra-mini‑PCNL or SWL. The complete stone clearance or presence of clinically insignificant residual fragments (<4 mm) 2 or 4 weeks after the final procedure was regarded as stone‑free. The presence of any fragments >4 mm after 4 weeks of treatment was considered as a residual stone needing further treatment.

Further treatment was in the form of another session of SWL combined with stone expulsive therapy and follow-up after another 2 weeks.

JJ was removed from the stented SWL group upon the clearance of sizable stones (no fragments larger than 4 mm).

### Statistical analysis

Recorded data were analysed using the Statistical Package for Social Sciences, version 23.0 (SPSS Inc., Chicago, Illinois, USA). The quantitative data are presented as mean ± standard deviation and ranges, while qualitative variables are presented as number and percentages.

## Results

Descriptive analysis of both groups regarding age, gender, stone size, location, HUs, laterality and number of shock waves is shown in Table 1.

**Table 1.** Descriptive analysis of both groups regarding age, gender, stone size, location, opacity, Hounsfield units, laterality and number of shock waves.

		Ultra-mini PCNL		Stented SWL*			
						Test value	*P*-value
Age	Mean ± SD	39.69 ± 12.83		38.85 ± 11.89		0.307•	0.759
	Range	20–66		21–60			
Gender	Female	15	35.7%	13	32.5%	0.094*	0.759
	Male	27	64.3%	27	67.5%		
Stone size	Mean ± SD	18.48 ± 1.80		18.80 ± 1.52		−0.878•	0.383
	Range	15–20		15–20			
Stone location	Upper calycael	2	4.8%	2	5.0%	3.996*	0.262
	Mid calycael	6	14.3%	4	10.0%		
	Lower calycael	9	21.4%	3	7.5%		
	Pelvic	25	59.5%	31	77.5%		
Stone opacity	Faint opaque	14	33.3%	12	30.0%	0.105*	0.746
	Radio opaque	28	66.7%	28	70.0%		
Hounsfield units	Mean ± SD	964.52 ± 211.23		989.98 ± 224.88		0.279•	0.599
	Range	640–1460		595–1425			
Laterality	Left	16	38.1%	16	40.0%	0.031*	0.860
	Right	26	61.9%	24	60.0%		
Number of shock waves				1300–3000			
				2546.25 ± 531.25			

*.Chi-square test; •: Independent *t*-test.

As for the SFR, there was increased frequency in the ultra-mini-PCNL group (37 patients (88.1%)) compared to the stented SWL group (32 patients (80%)), yet this difference did not pose any statistically significant difference with *P*-value being *P* = 0.316 (Table 2).

**Table 2.** Comparison between both groups regarding complications, fever, haematuria and need for blood transfusion and regarding stone-free rate and need for retreatment.

	Ultra-mini PCNL		Stented SWL		Chi-square test	
					*X*^2^	*P*-value
Fever	7	16.7%	5	12.5%	0.051	0.822
Haematuria	10	23.8%	6	15.0%	0.528	0.468
Need for blood transfusion	1	2.4%	0	0.0%	FE	0.326
Stone-free	37	88.1%	32	80.0%	1.006	0.316
Need for retreatment	5	11.9%	6	15.0%	0.169	0.681

Regarding the need for retreatments and residual stone >4 mm with auxiliary procedures, there was increased frequency in stented SWL group (6 patients (15%)) compared to the ultra-mini-PCNL group (5 patients (11.9%)), yet this difference was statistically insignificant with *P*-value being *P* = 0.681 (Table 2).

Although 16 patients developed haematuria postoperatively, 10 patients (23.8%) in PCNL group and 6 patients (15%) in SWL group, only 1 patient required blood transfusion postoperatively in the ultra-mini-PCNL group. As regarding postoperative fever, 12 patients developed fever, 7 of them (16.7%) in PCNL group and 5 patients (12.5%) in SWL group. Running chi-square test between the two groups, we found no statistically significant difference between both groups regarding post-operative complications with *P*-value being 0.822, 0.468 and 0.326, respectively, between both groups regarding fever, haematuria and need for blood transfusion (Table 2).

Regarding operative time, it ranged from 60 min to 115 min, with mean time of 89.83 ± 13.37 min in the ultra-mini PCNL group, while for the stented SWL group, it ranged from 20 min to 50 min, with mean time of 39.40 ± 8.78 min. There was highly statistically significant higher mean value in PCNL than SWL with *P*-value < 0.001 (Table 3).

**Table 3.** Comparison between both groups regarding operative time and hospital stay.

		Ultra-mini PCNL	Stented SWL		
				*t*-test	*P*-value
Operative time	Mean ± SD	89.83 ± 13.37	39.40 ± 8.78	31.04	<0.001
	Range	60–115	20–50		
Hospital stay	Mean ± SD	3.26 ± 0.45	1.18 ± 0.38	22.666	<0.001
	Range	3–4	1–2		

Hospital stay ranged from 3 to 4 days, with mean 3.26 ± 0.45 days in the ultra-Mini-PCNL group vs 1–2 days only with mean 1.18 ± 0.38 days, along the stented SWL group, showing a highly statistically significant difference between both groups with *P*-value being <0.001 (Table 3).

**Figure 2.**
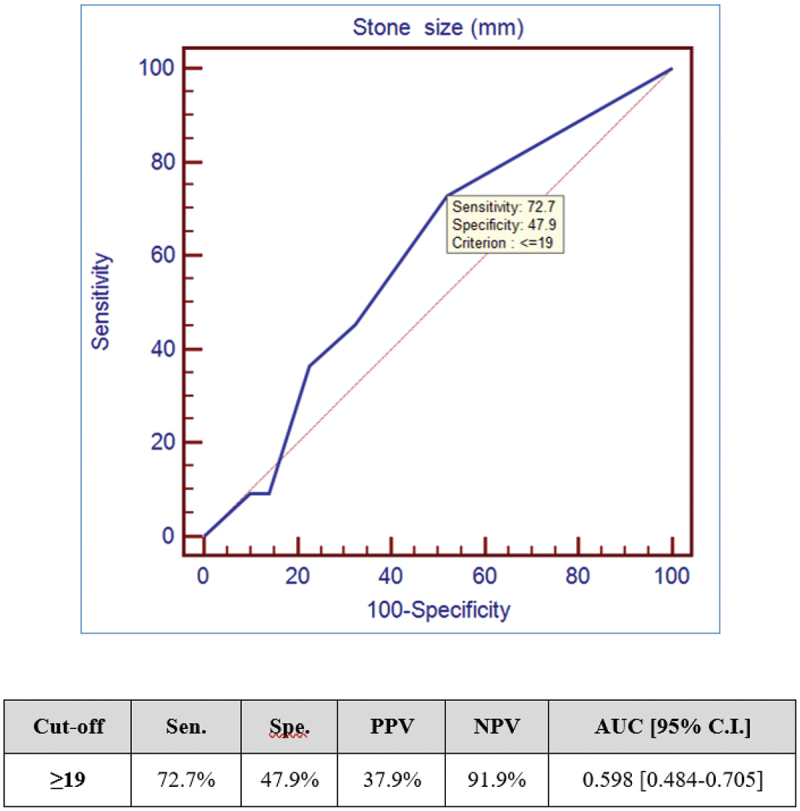
ROC curve and diagnostic accuracy of stone size in the prediction of the need for retreatment among study group.

Receiver operator characteristics (ROC) curves were indices of stone size as predictors of need for retreatment in included patients. Stone size indices were significant predictors; regarding the need for retreatment, it was used to define the best cut-off value of stone size which was ≥19, with sensitivity of 72.7%, specificity of 47.9%, positive predictive value of 37.9% and negative predictive value of 91.9% with diagnostic area under the curve of 0.598 [0.484–0.705] (Figure 2).

## Discussion

In our study, we found that SFR was slightly higher in the ultra-mini-PCNL group vs the stented SWL group yet without a statistically significant difference between the two groups. As for the need for retreatment, it was almost close among both groups, with no statistically significant difference (Table 3). However, ROC curves showed that stone size indices were significant predictor for the need for retreatment (Figure 2).

This was not like what Bozzini et al. stated in their study where they found that RIRS and PCNL were more effective than SWL in obtaining a stone-free condition, with a lower need for auxiliary procedures and retreatments with a reasonable rate of complications, but this may be attributed to that being directed to the choice of only lower calyceal stones only which was not of our inclusion criteria [6].

Also, Tsai et al. in their meta-analysis concluded that PCNL was associated with the best SFR for the management of Lower Pole Stones (LPS) regardless of age, sex and stone size. But again, this may be attributed to the choice of lower polar stones in their study [5].

Regarding complication rate, although one patient required blood transfusion in the ultra-mini-PCNL group, there was no statistically significant difference among both groups in the recorded complications regarding fever, haematuria and the need for blood transfusion (Table 2). This is also supported by Kim et al. in their study as they concluded that there is no reason to prefer SWL or RIRS over PCNL because of its complication rate. They declared that the complication rate, the largest disadvantage of PCNL, was relatively higher than those of SWL and RIRS when stone size was not considered, and they also stated that recent reports have outpointed the advantage of the less invasiveness of mini-PCNL and ultra-mini PCNL [2].

This was also found to be similar to what Tsai et al. found in their meta-analysis where they stated that there was no significant difference of complication, either overall complication rate or major complication rate, which was noted among RIRS, PCNL, mini-PCNL, micro-PCNL, SWL and conservative observation [5].

Regarding the operative time and hospitalization time, patients of the ultra-mini-PCNL group had longer operative time and longer hospital stay time compared to those of the stented SWL group (Table 3). Longer operative time may be related to the nature of the ultra-mini-PCNL procedure that requires some time in changing the position of the patient after the ureteric stent application. As for the longer hospital stay, it may be attributed to the presence of a nephrostomy tube that increased the hospital stay. This was similar to what has been stated by Zhang et al. in their study where they found that the ultra-mini-PCNL group had a longer hospitalization time than SWL and RIRS [10].

## Conclusion

Both stented SWL and ultra-mini-PCNL are reasonable treatment choices for renal stones sized less than 2 cm, with low complication rates. Denying ultra-mini-PCNL from patients with medium-sized stones for fear of complications is considered unwarranted. Stone size indices in our study were significant predictor for the need for retreatment, which brings us to the need for further studies to compare SFR based on stone size in both interventions.

## Limitations of our study

SWL session was done under fluoroscopy only due to the lack of the ultrasound probe at the time of conducting our study. All patients in the SWL group were stented in order to decrease the variables and to avoid the formation of steinstrasse which might require reoperation.

## Abbreviations list

Percutaneous nephrolithotomy: PCNL
Shock wave lithotripsy: SWL
Retrograde intrarenal surgery: RIRS
Stone-free rate: SFR
Body mass index: BMI
Urinary tract infection: UTI
Plain X-ray of the urinary tract: KUB
Hounsfield units: HUs
Haemoglobin: Hb
Blood urea nitrogen: BUN
Sodium: Na^+^
Potassium: K^+^
Statistical Package for Social Sciences, version 23.0: SPSS
Receiver operator characteristic: ROC
